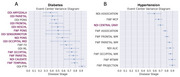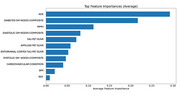# Brain Changes Associated with HbA1c and Systolic Blood Pressure Observed Using NODDI Contribute to Prediction of Cognition

**DOI:** 10.1002/alz70856_103713

**Published:** 2025-12-24

**Authors:** Xi Yu, Mingzhao Hu, Scott A. Przybelski, Robert I. Reid, Maria Vassilaki, Jonathan Graff‐Radford, Ronald Petersen, Clifford R. Jack, Prashanthi Vemuri, Sheelakumari Raghavan

**Affiliations:** ^1^ Mayo Clinic, Rochester, MN, USA; ^2^ Department of Quantitative Health Sciences, Mayo Clinic, Rochester, MN, USA; ^3^ Department of Neurology, Mayo Clinic, Rochester, MN, USA; ^4^ Department of Radiology, Mayo Clinic, Rochester, MN, USA

## Abstract

**Background:**

Chronic disease conditions lead to brain changes elevating dementia risk. We hypothesized that tissue microstructural changes caused by type 2 diabetes mellitus (T2DM) and hypertension could be effectively captured using an advanced diffusion MRI technique, Neurite Orientation Dispersion and Density Imaging (NODDI). Our goal was to evaluate the usefulness of composite NODDI scores, customized for increased hemoglobin (Hb) A1c and systolic blood pressure as predictors of cognition and compare their performance to other measures typically used to assess dementia risk.

**Method:**

We collected multi‐shell diffusion MR on 1024 Mayo Clinic Study of Aging participants. NODDI measures (neurite density index (NDI), orientation dispersion index (ODI), and free water fraction (FWF)) in gray matter (GM) and white matter (WM) regions were estimated. To identify brain regions susceptible to both conditions, a multiple linear regression model was fitted for each NODDI metric, using hemoglobin (Hb) A1c (in *N* = 305 participants) or systolic blood pressure (*N* = 906) as predictor, with age, sex, and body mass index (BMI) as covariates. Event‐based modeling was performed with the identified NODDI metrics to investigate the chronological pattern along the course of chronic condition development from this cross‐sectional dataset. Composite metrics were created as a weighted sum of susceptible NODDI metrics. We evaluated the impact of both conditions on cognition using gradient boosting models incorporating composite metrics, additional brain measurements, and demographics.

**Result:**

Increased HbA1c impacts both GM and WM NODDI relatively evenly, whereas increased systolic blood pressure related NODDI changes were mainly seen in WM (data not shown). With increased HbA1c, the earliest changes were observed in GM ODI, followed by changes in the pons and cortical FWF (Figure 1A). With increased systolic blood pressure, the earliest changes occurred in association fibers and middle cerebellar peduncle, followed by FWF change in lobar and projection WM fibers (Figure 1B). Average feature importance from 100 gradient boosting models for cognition prediction suggests that diabetes‐induced GM changes play a moderately important role in cognitive decline (Figure 2).

**Conclusion:**

Chronic medical conditions, especially T2DM, result in brain changes that are captured by NODDI and contribute moderately to cognitive performance.